# Perioperative Anesthetic Challenges and Multidisciplinary Management of a Massive Intra-Abdominal Tumor

**DOI:** 10.14740/jmc5363

**Published:** 2026-07-28

**Authors:** Majlinda Naco, Dorina Ruci Serani, Rudin Domi, Gentian Huti, Asead Abdyli, Burak Koza, Driola Hoxha, Krenar Lilaj, Hektor Sula, Alma Cani, Filadelfo Coniglione, Migena Vargu, Vedat Eljezi

**Affiliations:** a Department of Surgery, Service of Anesthesia and Intensive Care, University of Medicine, Tirana, Albania; b Department of Internal Medicine, Service of Rheumatology, University of Medicine, Tirana, Albania; c Department of Anesthesia and Intensive Care, American Hospital 3, Tirana, Albania; d Department of Surgery, American Hospital 3, Tirana, Albania; e Department of Clinical Science and Translational Medicine, Tor Vergata University of Rome, Rome, Italy; f Department of Infectious Diseases, Service of Dermatology, University of Medicine, Tirana, Albania; g Department of Perioperative Medicine, CHU Gabriel-Montpied, Clermont-Ferrand, France

**Keywords:** Giant ovarian tumor, Anesthesia, Perioperative management, Intra-abdominal mass, Intra-abdominal pressure

## Abstract

Massive intra-abdominal tumors are associated with major perioperative anesthetic and surgical challenges due to respiratory compromise, hemodynamic instability, difficult positioning, fluid shifts, and postoperative complications. We present the case of a 65-year-old woman with a giant ovarian tumor weighing approximately 21 kg who underwent successful surgical resection following multidisciplinary perioperative management. A massive abdominal cystic lesion occupying most of the abdominal cavity, compressing abdominal organs, and associated with thoracic abnormalities was identified. Surgical treatment included hysterectomy, omentectomy, appendectomy, and cystectomy. The novelty of this case lies in the extreme tumor size, the severe positional hemodynamic instability caused by inferior vena cava compression, and the intraoperative confirmation of increased intra-abdominal pressure requiring immediate adaptation of anesthetic and surgical management. Despite the complexity of the case, the patient had a favorable perioperative course. This case highlights the importance of careful anesthetic planning, respiratory optimization, invasive monitoring, and multidisciplinary collaboration in the management of giant abdominal tumors.

## Introduction

Giant abdominal tumors are rare lesions originating from the abdominal wall, intra-abdominal cavity, or retroperitoneal space, usually defined by a diameter greater than 15 cm or a weight exceeding 2 kg [1]. Although advances in imaging techniques and earlier diagnosis have reduced their incidence in modern clinical practice, giant ovarian tumors continue to represent major perioperative challenges for both surgeons and anesthesiologists.

Because of their massive size, these tumors may severely affect respiratory and cardiovascular function through compression of the diaphragm and major abdominal vessels. Patients can develop reduced pulmonary compliance, impaired venous return, respiratory compromise, and supine hypotension before surgery. In addition, rapid decompression after tumor removal may lead to abrupt hemodynamic changes, cardiovascular collapse, or re-expansion pulmonary edema [2]. Giant intra-abdominal masses may also cause metabolic disturbances, difficult patient positioning, major intraoperative fluid shifts, and challenging ventilatory management [3].

Current evidence regarding anesthetic management of giant abdominal tumors remains limited, with most available literature consisting of isolated case reports and small case series. These tumors may produce profound physiological disturbances, including impaired respiratory mechanics, reduced venous return, intra-abdominal hypertension, and abrupt cardiovascular changes during surgical decompression, making perioperative management particularly challenging. Consequently, individualized anesthetic approaches and close multidisciplinary coordination are fundamental to ensure perioperative stability and optimize patient outcomes.

Although several cases of giant abdominal tumors have been reported, this case contributes additional evidence by describing the perioperative management of an exceptionally large intra-abdominal mass associated with severe physiological derangements and substantial anesthetic challenges. Its novelty lies in the detailed presentation of the multidisciplinary strategy, including preoperative optimization, invasive hemodynamic monitoring, tailored ventilatory support, and careful management during tumor decompression and resection. By emphasizing the anesthetic decision-making process and dynamic physiological changes, this report provides practical lessons for managing these rare and complex cases, for which evidence remains limited.

## Case Report

### Investigations

**Figure 1:**
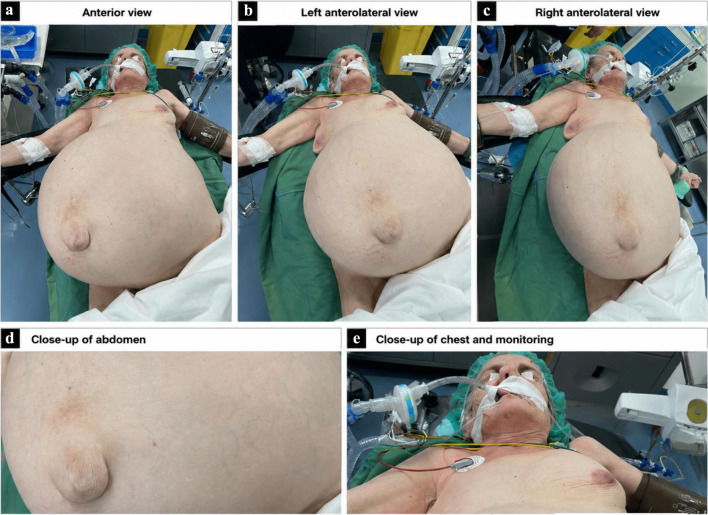
A giant tumor caused marked abdominal distension and increased intra-abdominal pressure.

**Figure 2:**
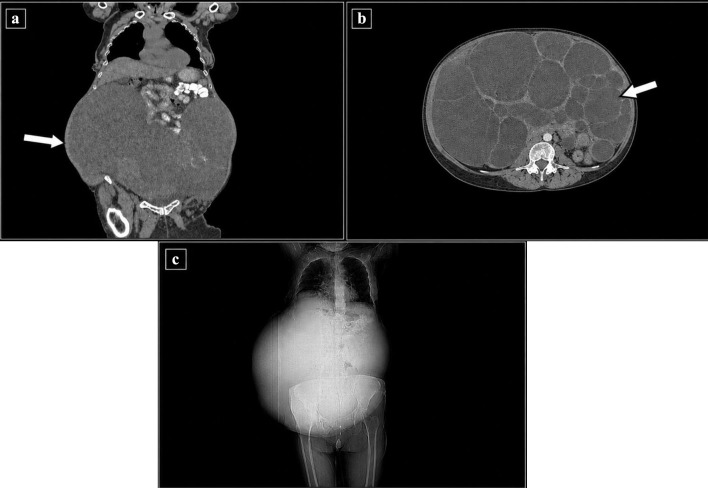
Computed tomography (CT) scan examination revealed large intra-abdominal tumor. (a) Coronal contrast-enhanced CT image showing a large intra-abdominal tumor occupying the lower abdomen. Arrow indicates giant tumor. (b) Axial contrast-enhanced CT image demonstrating the multi-cystic nature of the tumor. Arrow indicates cysts. (c) CT scout image demonstrating the marked abdominal distention caused by the large intra-abdominal mass.

A 65-year-old woman (68 kg, 161 cm; body mass index (BMI) 26) was admitted for surgical resection of a massive intra-abdominal tumor. Six years before admission, an intra-abdominal mass had been detected; however, no intervention was performed because the patient refused further treatment and follow-up. Over time, the abdominal mass progressively increased in size and became associated with marked abdominal distension and functional limitation (Fig. 1). One month before surgery, the patient was hospitalized because of pneumonia. During this admission, additional diagnostic evaluation was performed, including abdominal ultrasonography and angio-abdominal computed tomography (CT) scan (Fig. 2). Imaging confirmed the presence of a massive multinodular cystic intra-abdominal tumor occupying most of the abdominal cavity. Fine-needle aspiration was subsequently performed, and pathological evaluation was obtained for further characterization of the lesion. Previous medical history was unremarkable, with no chronic medical diseases reported, except for thyroid gland nodules treated with propranolol. Thyroid function tests, including thyroid hormones and thyroid-stimulating hormone (TSH), were within normal preoperative ranges. Preoperative respiratory evaluation, including clinical examination and respiratory function testing, demonstrated values within normal limits. Preoperative laboratory investigations were largely within normal limits. The patient had experienced pneumonia 1 month before surgery, with residual respiratory findings noted on thoracic imaging.

### Diagnosis

CT of the thorax and abdomen demonstrated a giant cystic formation measuring approximately 40 × 20 × 40 cm, associated with minimal pleural effusion, air trapping, and mosaic perfusion abnormalities in the lungs. Tumor marker evaluation revealed a CA-125 level of 3,732 U/mL (reference range 0–35 U/mL), raising concern for ovarian malignancy. Ascitic fluid analysis demonstrated glucose < 5 mg/dL, protein 9.2 g/dL, lactate dehydrogenase (LDH) 3,280 U/L, neutrophils 80%, lymphocytes 17%, and eosinophils 3%. Ascitic fluid analysis demonstrated an exudative inflammatory profile with markedly elevated protein and LDH levels, severe glucose depletion, and neutrophil predominance, findings suggestive of malignant peritoneal involvement.

### Treatment

**Figure 3:**
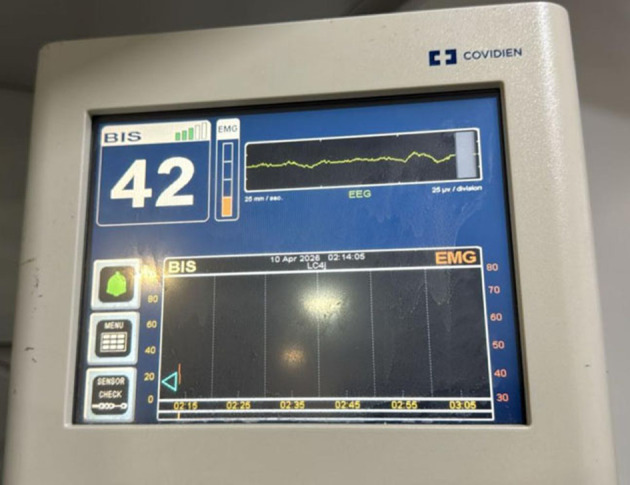
Bispectral Index (BIS) monitoring showed a value of 42, indicating an adequate depth of anesthesia during the procedure and helping guide anesthetic titration despite significant intraoperative hemodynamic fluctuations.

The patient underwent exploratory laparotomy under general anesthesia. Given the giant tumor size and the risk of hemodynamic instability, careful anesthetic induction and invasive hemodynamic monitoring were implemented. Anesthesia was induced with propofol 150 mg, vecuronium 6 mg, and fentanyl 150 mcg. Total intravenous anesthesia (TIVA) was maintained using propofol 75 µg/kg/min and remifentanil 0.3 µg/kg/min, guided by Bispectral Index (BIS) monitoring (Fig. 3).

**Figure 4:**
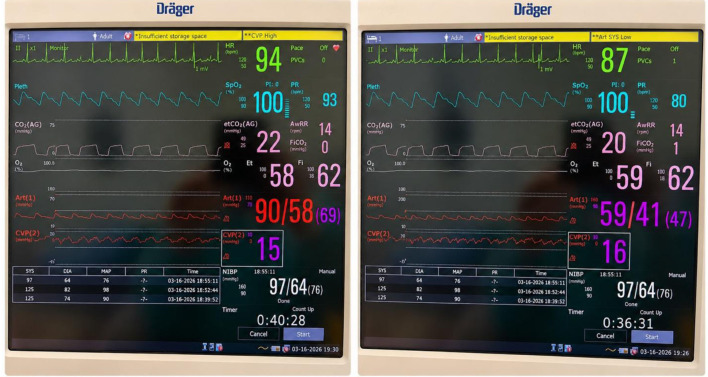
Hemodynamic parameters during different intraoperative phases (a) and hypotension (ART 1 red wave) occurred only by patient’s abdominal repositioning (b). In both monitoring views central venous pressure (CVP, red wave) is high, indicating intra-abdominal pressure.

During patient positioning, severe hypotension occurred due to compression of the inferior vena cava by the massive tumor (Fig. 4). The patient’s position was subsequently adjusted, requiring modification of the surgical approach. The intra-abdominal pressure (IAP), measured indirectly via urinary bladder pressure, was 16–18 mm Hg, confirming the presence of grade II intra-abdominal hypertension.

**Figure 5:**
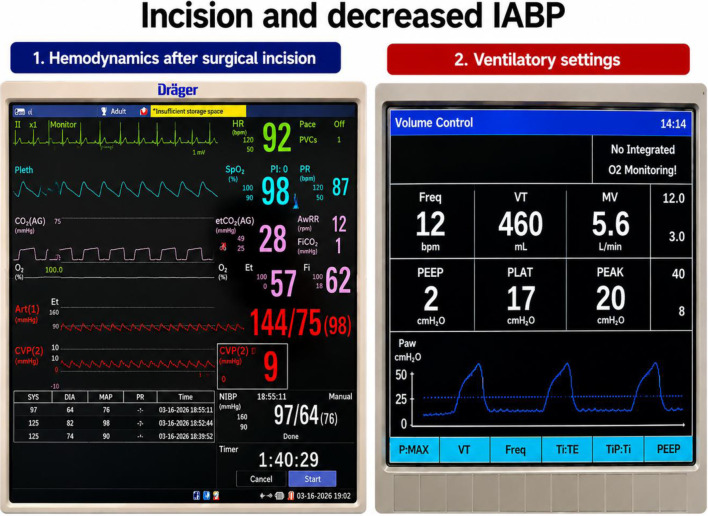
Hemodynamic and ventilatory parameters improved following surgical incision and relief of intra-abdominal pressure. HR: heart rate; SpO_2_: pulse oximetry; ETCO2: end-tidal CO_2_; AwRR: airway respiratory rate; Art: arterial pressure; CVP: central venous pressure; NIBP: non-invasive blood pressure; PI: Perfusion Index; PR: pulse rate; PVCs: premature ventricular contractions; VT: tidal volume; MV: minute ventilation; PEEP: positive end-expiratory pressure; PLAT: plateau pressure; PEAK: peak inspiratory pressure; Paw: airway pressure; Ti: inspiratory time; TE: expiratory time; TiP: pause time.

After surgical incision and release of the IAP, both hemodynamic and ventilatory parameters markedly improved (Fig. 5). Lung-protective mechanical ventilation strategies were applied to minimize respiratory compromise related to increased IAP and reduced pulmonary compliance. Intraoperative ventilation included a tidal volume of 6 mL/kg, plateau pressure less than 30 cm H_2_O, fraction of inspired oxygen (FiO_2_) 0.5, and positive end-expiratory pressure (PEEP) of 5–8 cm H_2_O, maintaining normal intraoperative pH, PaO_2_, and PaCO_2_ levels while reducing the risk of postoperative pulmonary complications.

Invasive arterial blood pressure and central venous pressure (CVP) monitoring were established, including central venous catheterization via the internal jugular vein. Hemodynamic stability was maintained with Ringer’s lactate, albumin, packed red blood cells (PRBCs), and noradrenaline infusion at 0.02–0.08 µg/kg/min. Intraoperatively, the patient received 3,500 mL of Ringer’s lactate, two 50 mL doses of 20% albumin, and two units of PRBCs. Table 1 summarizes intraoperative hemodynamic, respiratory, and metabolic data.

**Table 1:** Perioperative Hemodynamic Changes and Intraoperative Clinical Data Associated With Giant Intra-Abdominal Tumor Resection

Variable	Before mass removal	After mass removal
Arterial blood pressure (mm Hg)	59/41	Improved with vasopressor and fluid therapy^a^
CVP (mm Hg)	16	1
Peak inspiratory pressure (cm H_2_O)	34	20
Plateau pressure (cm H_2_O)	29	17
Urinary output	2,000 mL	
Estimated blood loss	200 mL	
Ringer’s lactate administered	3,500 mL	
Hemoglobin	9.5 g/dL	
Lactate	1.3 mmol/L	
Time to extubating	2 h after ICU admission	

^a^Hemodynamic stability was achieved with crystalloid administration and noradrenaline infusion under invasive monitoring. CVP: central venous pressure.

**Figure 6:**
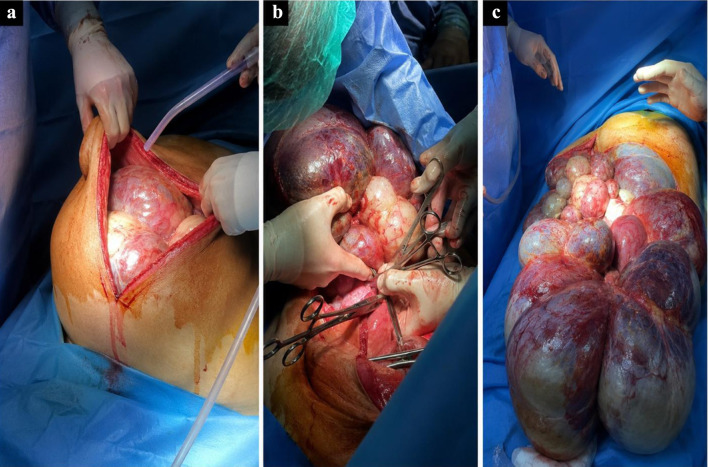
Different surgical stages, from the surgical incision (a) and preparation of the anatomical resection plan (b) to complete removal of the giant tumor (c).

The surgical procedure included not only tumor resection, but also hysterectomy, omentectomy, appendectomy, and cystectomy, with a total operative time of approximately 5 h (Fig. 6).

Following surgery, the patient remained intubated and was transferred to the intensive care unit (ICU) for postoperative monitoring and subsequent extubating. Postoperative analgesia was achieved with a multimodal, opioid-sparing regimen based on nonsteroidal anti-inflammatory drugs (NSAIDs). This approach provided satisfactory pain control, facilitated effective respiratory function and early mobilization, and minimized the risk of opioid-related adverse effects, thereby contributing to an uneventful postoperative recovery.

### Follow-up and outcomes

The postoperative course was stable, and the patient was transferred for continued monitoring and supportive care in ICU. The next day the patient was discharged in ward. Histopathological examination confirmed the diagnosis of bilateral low-grade papillary serous cystadenocarcinoma of the ovaries. Macroscopic evaluation revealed giant multi cystic ovarian masses with serous and mucinous content, associated with focal solid areas and extensive dystrophic calcifications. Microscopic analysis demonstrated stromal invasion and multifocal omental infiltration by neoplastic tissue. Additional tumor involvement was identified in the right and left broad ligaments, as well as peritoneal nodules, while the appendix was free of neoplastic infiltration. Cytological examination revealed papillary atypical cellular groups consistent with carcinomatous dissemination. The final pathological staging was reported as pT3cNxMx (FIGO stage IIIc), and molecular testing for KRAS and BRAF mutations was recommended. The patient received adjuvant chemotherapy consisting of carboplatin and paclitaxel every 3 weeks for a total of six cycles. After the first three cycles, serum tumor markers normalized, and abdominal ultrasonography revealed no evidence of residual or recurrent disease. Upon completion of the six chemotherapy cycles, a CT scan will be performed to evaluate the treatment response and exclude disease recurrence. If the postoperative and oncological course remains uneventful, the patient will undergo regular surveillance consisting of serum tumor marker assessment every 3 months, complemented by periodic imaging studies according to clinical indications and institutional follow-up protocols.

## Discussion

### Hemodynamic challenges

Large abdominal masses may compromise venous return and cardiac output, particularly during induction of anesthesia and tumor decompression. Sudden removal of compressive forces can result in rapid hemodynamic changes and fluid redistribution; therefore, invasive monitoring and meticulous fluid management are essential.

Previous reports of giant ovarian and intra-abdominal tumors have described severe hemodynamic disturbances resulting from compression of the inferior vena cava, aorta, and diaphragm, leading to reduced preload, decreased cardiac output, and hypotension [4, 5]. Similar physiological alterations have also been observed during pneumoperitoneum, where elevated IAP reduces venous return and increases systemic vascular resistance [6–8]. Although our patient did not undergo laparoscopy, these studies are clinically relevant because they demonstrate the cardiovascular consequences of increased IAP, which were also present in our case.

In contrast to many reported cases in which hemodynamic instability mainly occurred after induction or during tumor removal [4, 5], our patient developed profound hypotension during positioning before surgical incision. We believe this event was primarily related to severe inferior vena cava compression by the massive tumor, further aggravated by changes in body position. This finding influenced our management strategy, prompting immediate repositioning, invasive reassessment, and modification of the surgical approach before proceeding with definitive decompression.

The measurement of bladder pressure confirmed significant intra-abdominal hypertension and supported our hypothesis that elevated IAP was a major contributor to both circulatory and respiratory impairment. Based on the pathophysiological principles described by Weinberg et al and studies evaluating increased IAP [5–8], we opted for continuous arterial pressure monitoring, central venous access, cautious fluid administration, and early vasopressor support to maintain adequate organ perfusion while avoiding excessive fluid loading.

Unlike reports describing preoperative cyst drainage as a means of reducing hemodynamic complications [4], this approach was not feasible in our patient because of the tumor characteristics and the need for definitive surgical resection. Instead, controlled surgical decompression combined with continuous hemodynamic monitoring allowed prompt recognition of physiological changes. Following abdominal decompression, both cardiovascular and ventilatory parameters improved markedly, confirming that mechanical compression and intra-abdominal hypertension were the principal determinants of the patient’s instability. Subsequently, hemodynamic stability was maintained with individualized administration of crystalloids, albumin, PRBCs, and noradrenaline infusion under invasive monitoring.

### Respiratory challenges

Intra-abdominal hypertension and abdominal compartment syndrome significantly affect respiratory function by reducing lung compliance and functional residual capacity, increasing intrathoracic pressure and airway pressures, and impairing gas exchange [9, 10].

Several reports of giant intra-abdominal tumors have described similar respiratory derangements and have emphasized strategies such as reverse-Trendelenburg positioning, alveolar recruitment maneuvers, PEEP application, and gradual decompression to improve oxygenation and pulmonary mechanics [11–13]. Studies evaluating pneumoperitoneum have likewise demonstrated that increased IAP leads to elevated airway pressures, reduced pulmonary compliance, hypercapnia, and postoperative pulmonary complications [14, 15].

Our patient exhibited many of these pathophysiological features, including reduced diaphragmatic excursion, impaired thoracoabdominal mechanics, and decreased pulmonary compliance caused by the massive tumor burden and increased IAP. However, unlike previously reported cases, respiratory compromise was further aggravated by residual pulmonary abnormalities following recent pneumonia, substantially increasing the risk of perioperative hypoxemia and postoperative respiratory complications.

These considerations guided our anesthetic strategy. Because elevated IAP was considered the principal mechanism of respiratory impairment, we adopted a lung-protective ventilation approach consisting of low tidal volume ventilation, optimization of PEEP, limitation of plateau pressures, and continuous monitoring of gas exchange. The use of PEEP was supported by previous studies demonstrating its beneficial effects on alveolar recruitment and prevention of postoperative pulmonary complications [16, 17], although its application in our patient required careful titration to avoid further reduction in venous return.

A particularly important observation in our case was the immediate improvement in ventilatory parameters after abdominal decompression. This response strongly suggested that increased IAP and mechanical diaphragmatic compression were the dominant causes of respiratory dysfunction. Consequently, our experience supports previous observations that early recognition of abdominal hypertension and individualized ventilatory management are essential in patients with giant intra-abdominal tumors [11–15]. Moreover, it highlights the importance of integrating respiratory and hemodynamic management, as interventions aimed at improving pulmonary mechanics may substantially influence cardiovascular stability in these complex patients.

### Postoperative care

Postoperative management following resection of a giant intra-abdominal tumor requires intensive monitoring because these patients are at high risk of significant physiological disturbances. Hemodynamic instability may persist after surgery due to abrupt changes in venous return, redistribution of blood volume, residual effects of prolonged organ compression, and ongoing fluid shifts into the previously compressed abdominal cavity [18]. Therefore, admission to the ICU is often warranted for continuous monitoring of arterial pressure, heart rate, urine output, and serial laboratory investigations, including hemoglobin, electrolytes, lactate, and coagulation parameters. Careful titration of intravenous fluids and vasoactive agents may be necessary to maintain adequate organ perfusion while avoiding fluid overload. Goal-directed hemodynamic management has been associated with improved postoperative recovery and reduced complications following major abdominal surgery [19].

Respiratory management is another critical component of postoperative care. Following removal of a massive abdominal mass, the diaphragm and chest wall undergo significant mechanical changes, potentially resulting in atelectasis, pulmonary edema, or respiratory failure [20]. Patients with prolonged preoperative diaphragmatic elevation and restrictive physiology may require delayed extubation and postoperative mechanical ventilation until adequate respiratory mechanics and gas exchange are achieved. Aggressive pulmonary care, including early physiotherapy, incentive spirometry, effective analgesia, and early mobilization, is essential to reduce postoperative pulmonary complications [20, 21].

Additionally, postoperative surveillance should focus on early detection of complications related to extensive tumor resection, including bleeding, abdominal compartment syndrome, electrolyte disturbances, acute kidney injury, and thromboembolic events [22]. Adequate multimodal analgesia facilitates respiratory function and early mobilization while minimizing opioid-related adverse effects [23].

Compared with previously reported cases of giant intra-abdominal tumor resection, our case exhibited similar perioperative concerns related to the risk of abrupt hemodynamic and respiratory changes following tumor decompression. However, despite the massive size of the tumor and significant intraoperative hemodynamic fluctuations, the postoperative course was favorable, without major respiratory or cardiovascular complications. The patient was successfully managed with careful postoperative monitoring, appropriate analgesia, and early supportive care. This favorable outcome underscores the importance of meticulous perioperative planning and a multidisciplinary approach in mitigating the physiological consequences associated with the removal of giant intra-abdominal masses.

### Conclusion

Massive intra-abdominal tumors pose unique anesthetic challenges because of their profound effects on cardiovascular and respiratory physiology. Increased IAP and tumor compression can precipitate severe hemodynamic instability, impaired venous return, reduced lung compliance, and perioperative respiratory failure, particularly during induction and surgical decompression. Safe perioperative management requires meticulous planning, invasive monitoring, individualized hemodynamic support, and lung-protective ventilation strategies, with anticipation of rapid physiological changes following tumor removal. A multidisciplinary approach and continuous reassessment remain essential to optimize outcomes and minimize perioperative morbidity in these complex patients.

### Learning points

Giant ovarian tumors can cause severe respiratory and hemodynamic compromise due to diaphragmatic and vascular compression.

Careful multidisciplinary perioperative planning is essential for successful management of massive intra-abdominal tumors.

Inferior vena cava compression during positioning may trigger profound hypotension requiring immediate positional adjustment.

Lung-protective ventilation, invasive monitoring, and optimized fluid-vasopressor therapy are crucial during tumor resection.

Postoperative ICU monitoring is recommended because of the risk of delayed respiratory and hemodynamic complications.

## Data Availability

The authors declare that data supporting the findings of this study are available within the article.
